# A Pull All Together

**Published:** 1890-10

**Authors:** 


### EDITORIAL DEPARTMENT.

F. E. DANIEL, M. D., EDITOR AND PUBLISHER.

THIS JOURNAL, by unanimous vote of the members, was made the Official Organ of the Austin District Medical Society.

COLLABORATORS:

*Dr. R. M. Swearingen, Austin.*
*Prof. B. E. Hadra, M. D., Galveston.*
*Prof. Geo. Cupples, M. D., San Antonio.*
*Dr. T. C. Osborn, Cleburne, Texas.*
*Dr E. J. Doering, Chicago.*
*Dr. E. J. Beall, Fort Worth.*
*Dr Odo Betz, Germany.*
*Prof. W. B. Rogers, M. D., Memphis.*
*Dr. J. W. McLaughlin, Austin.*
*Dr. T. J. Tyner, Austin.*
*Prof. J. F. Y. Paine, M. D., Galveston.*
*Dr. R. H. L. Bibb, Mexico.*
*Dr. T. J. Bennett, Austin.*
*Dr. Bat Smith, Wharton.*
*Dr. E. Meierhof, New York.*
*L. H. Luce, M. D., West Tisbury, Mass.*

### A PULL ALL TOGETHER.

THE JOURNAL is much gratified to observe and announce that the Committee on Medical Legislation has adopted and carried out to the letter the plan outlined in our last issue, to secure concert of action in the matter of asking for medical legislation,—the establishment of a State Board of Health, a law to regulate the practice, etc.

It will be seen by the circular letter to the profession, published elsewhere in this issue, that the Committee have forcibly set forth the reasons why such legislation is needed and asked for; and that they have adopted the JOURNAL'S suggestion of sending petitions to all the local societies, to be signed by their members, and bv other reputable physicians, not members; it is also contemplated to secure the siguatures of a large number of influential laymen; and when *six thousand* petitions, each signed by two hundred or two hundred and fifty citizens and physicians are presented to the Legislature,—each one through the representative of the district whence it emanates,—it will certainly open the eyes of the legislators to the fact that the question is a living and vital issue, and no longer to be "pooh"-"poohed," and called "class legislation;" they will see that the *people* begin to appreciate the fact that while even their cattle have State protection, their families have none whatever in the matter of life and health.

*Salus populi est suprema lex*—is a proverb which, to the present time, has been fearfully parodied in Texas; the health of the people has been more neglected than the cattle on the plains,—but *nous allons changer tout cela;* and hereafter it is to be hoped Texas may, like the Romans of old, truthfully boast that the welfare and prosperity of the people constitute the supremest law, and they shall be guarded henceforth with jealous care. Great is Texas—in all save sanitary science;—and she is being awakened to its vast importance through the agency of the State Medical Association, voiced by this JOURNAL.

It can be demonstrated that a State Board of Health, such as exists in other States, can be operated at less *cost* to the State than our present quarantine system in time of no invasions. There have been no epidemics threatening even—and yet the quarantine expense has been more than that of the average Board of Health. True economy demands the establishment of a good board at the earliest possible moment, and the enactment of a law which shall limit the privilege of dealing with life and death—practicing medicine—to the hands of those who have been trained and educated in medical science.

